# High-Performance Ionanofluids from Subzipped Carbon Nanotube Networks

**DOI:** 10.1021/acsami.2c14057

**Published:** 2022-11-04

**Authors:** Marzena Dzida, Sławomir Boncel, Bertrand Jóźwiak, Heather F. Greer, Mateusz Dulski, Łukasz Scheller, Adrian Golba, Rafał Flamholc, Grzegorz Dzido, Justyna Dziadosz, Anna Kolanowska, Rafał Jędrysiak, Anna Blacha, Krzysztof Cwynar, Edward Zorębski, Carlos E.S. Bernardes, Maria José V. Lourenço, Carlos A. Nieto de Castro

**Affiliations:** † Institute of Chemistry, University of Silesia in Katowice, Szkolna 9, Katowice 40-006, Poland; ‡ Department of Organic Chemistry, Bioorganic Chemistry and Biotechnology, Silesian University of Technology, Bolesława Krzywoustego 4, Gliwice 44-100, Poland; § Centre for Organic and Nanohybrid Electronics, Silesian University of Technology, Konarskiego 22B, Gliwice 44-100, Poland; ∥ Department of Chemical Engineering and Process Design, Silesian University of Technology, Marcina Strzody 7, 44-100 Gliwice, Poland; ⊥ Department of Chemistry, University of Cambridge, Cambridge CB2 1EW, U.K.; # Faculty of Science and Technology, Institute of Materials Science, University of Silesia in Katowice, 75 Pułku Piechoty 1a, Chorzów 41-500, Poland; ¶ Anton Paar Poland, Hołubcowa 123, Warsaw 02-854, Poland; ∇ Department of Physical Chemistry and Technology of Polymers, Silesian University of Technology, Marcina Strzody 9, Gliwice 44-100, Poland; ○ Centro de Química Estrutural, Institute of Molecular Sciences, Departamento de Química e Bioquímica, Faculdade de Ciências, Universidade de Lisboa, Campo Grande, Lisboa 1749-016, Portugal

**Keywords:** ionanofluids, subzipping mechanism, “cobra-like” macromolecular architectures, thermal conductivity, viscosity

## Abstract

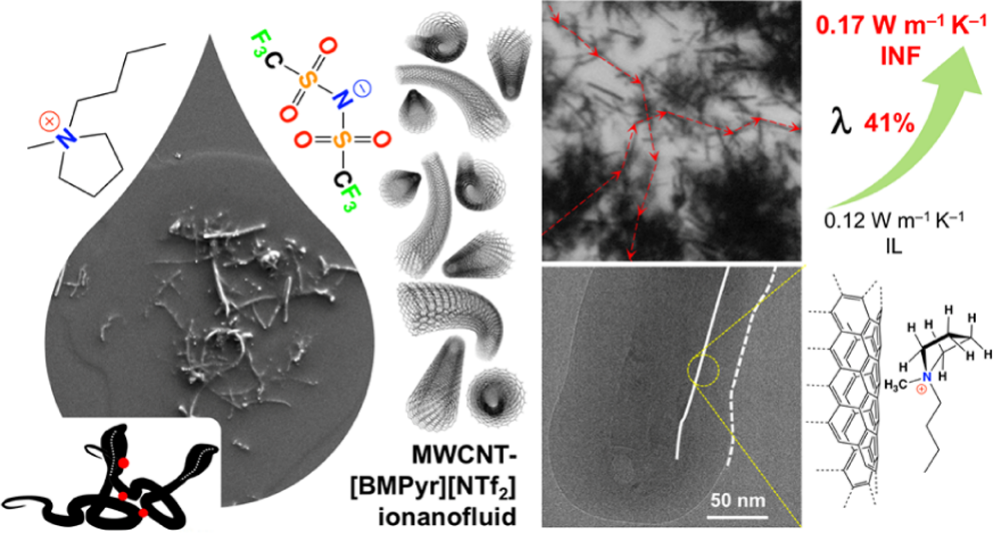

Investments in the transfer and storage of thermal energy along with renewable energy sources strengthen health and economic infrastructure. These factors intensify energy diversification and the more rapid post-COVID recovery of economies. Ionanofluids (INFs) composed of long multiwalled carbon nanotubes (MWCNTs) rich in sp^2^-hybridized atoms and ionic liquids (ILs) display excellent thermal conductivity enhancement with respect to the pure IL, high thermal stability, and attractive rheology. However, the influence of the morphology, physicochemistry of nanoparticles and the IL–nanostructure interactions on the mechanism of heat transfer and rheological properties of INFs remain unidentified. Here, we show that intertube nanolayer coalescence, supported by 1D geometry assembly, leads to the subzipping of MWCNT bundles and formation of thermal bridges toward 3D networks in the whole INF volume. We identified stable networks of straight and bent MWCNTs separated by a layer of ions at the junctions. We found that the interactions between the ultrasonication-induced breaking nanotubes and the cations were covalent in nature. Furthermore, we found that the ionic layer imposed by close MWCNT surfaces favored enrichment of the *cis* conformer of the bis(trifluoromethylsulfonyl)imide anion. Our results demonstrate how the molecular perfection of the MWCNT structure with its supramolecular arrangement affects the extraordinary thermal conductivity enhancement of INFs. Thus, we gave the realistic description of the interactions at the IL–CNT interface with its (super)structure and chemistry as well as the molecular structure of the continuous phase. We anticipate our results to be a starting point for more complex studies on the supramolecular zipping mechanism. For example, ionically functionalized MWCNTs toward polyionic systems—of projected and controlled nanolayers—could enable the design of even more efficient heat-transfer fluids and miniaturization of flexible electronics.

## Introduction

**Figure 1:**
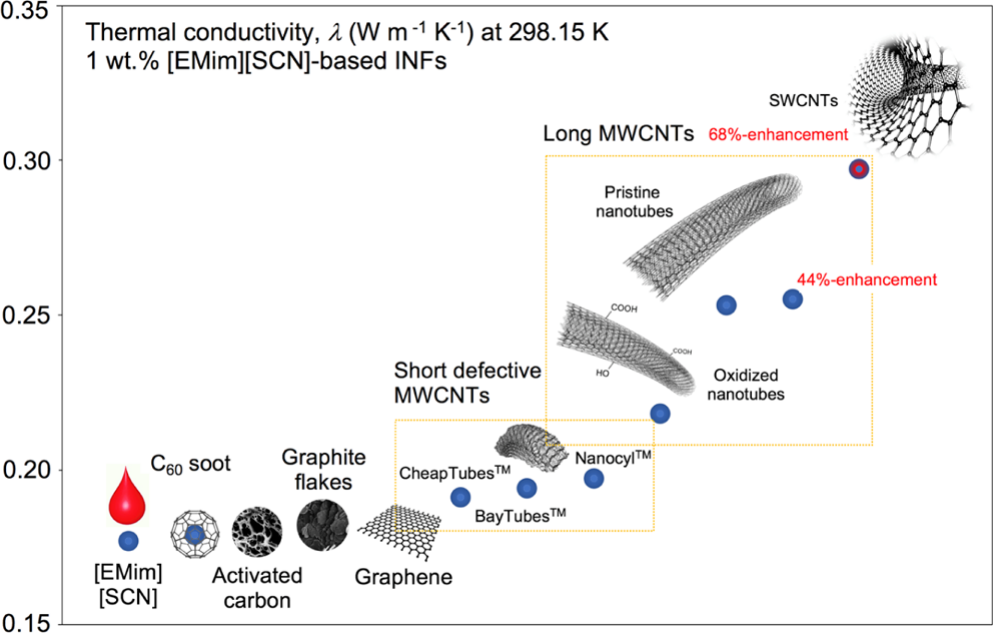
Impact of the morphology of carbon nanomaterials on the thermal conductivity of [EMim][SCN]-based INFs at 298.15 K (data from ref (4)).

Strengthening of the economic and healthcare infrastructure relies on the effort put in advancement of thermal energy transfer and storage, which, combined with renewable energy sources, leads to highly desirable energy diversification, as well as successful recovery of the post-COVID economy.^1,2^ By 2030, the market for heat-transfer fluids is predicted to be 7 billion USD.^3^ Ionanofluids (INFs)—derived from ubiquitous MWCNTs and ionic liquids (ILs)—represent modern systems of synergetic multifunctionality. These characteristics cover high thermal conductivity, nonflammability, and stability, which lead to efficient and safe heat-transfer media. To date, substantial efforts have been focused on the macroscopic response of INFs upon the addition of nanoparticles to ILs and the role of interfacial nanolayers on nanoparticles in terms of the molecular-level understanding of thermophysics.^4−6^ In contrast, the role of the CNT morphology, the underlying mechanisms of interactions at the IL–CNT interface with the CNT structure, and the molecular structure of continuous phases remain elusive. Thus, the Holy Grail in the INFs is their molecular design, including local, global, and multiscale descriptors. Recently, we studied the impact of the carbon nanomaterial morphology on thermal conductivity (Figure 1) and rheological characteristics of INFs based on the 0D fullerene soot, 1D MWCNTs and single-walled carbon nanotubes (SWCNTs), 2D graphene sheets, 3D graphite flakes, and activated carbon.^4^ Most importantly, we received a substantial increase in the thermal conductivity of 44% for INFs composed of long MWCNTs.^4^

We also found that the thermal conductivity of INFs based on SWCNTs yielded excellent enhancement in thermal conductivity of 68%, but even low SWCNT loadings produced “bucky gels” or high-viscous dispersions.^4^ Therefore, herein focusing our studies, we chose long MWCNTs with an originally high aspect ratio of 11,000, and—as the reference system—commercially available, short and defective MWCNTs with a moderate aspect ratio of 150. The rationale behind the selection of IL was also twofold. First, the nonaromatic (hence disabling π–π interactions) pyrrolidinium cation [BMpyr]^+^ was chosen to reduce the number of variables and to allow accurate charge referencing.^7^ Second, [NTf_2_]^−^ was selected because it is a relatively large, complex anion with a delocalized negative charge along the S–N–S ion core, and the steric hindrance reduces the number of ion–ion interactions.

Consecutively, the aim of this paper is to deal with the following two questions: first, how the morphology and physicochemistry of nanostructures, and, second, how the IL–nanostructure interactions, influence the mechanism of heat transfer and change the rheological properties of INFs. When addressing them, we found—in the whole INF volume—a partial subzipping of long MWCNT networks and thermal bridges of high intrinsic thermoconductivity within the 3D network. Crucially, the subzipping is understood here as the interactions between two neighboring nanotubes which are fragmentarily coalescent by the IL nanolayers, that is, intertube zipping together with locally unzipped individual pairs of nanotubes and/or longitudinally unzipped nanotubes. Such a geometry-driven, π–π stacking-based assembling enables the formation of stable thermal bridges between long MWCNTs prearranged via the self-sorting mechanism. We show that the first-contact molecular forces in the nanotube–IL interface strongly depend on the nanotube morphology in contrast to the interfacial geometry of IL nanolayers. For the C-sp^2^-rich, long, crystalline MWCNTs, we have identified the key role of IL in the sonication-induced subzipping, controlled nanotube cutting, and entrapment of *in situ*-formed nanotube dangling bonds. Thus, we present for the first time, structural and spectroscopic studies together with molecular dynamics (MD) simulations of interactions at the IL–CNT interface as well as the molecular structure of the bulk phase, which all give the realistic portrait of INFs.

## Experimental Section

### Materials

#### Multiwalled CNTs and IL

Long MWCNTs were synthesized in our laboratory via a 16 h catalytic chemical vapor deposition (c-CVD) process. Details were described in our previous papers.^4,8^ Short MWCNTs were purchased from Nanocyl, Belgium. Characteristics of the MWCNTs used in this study are summarized in Table 1. The base fluid in INFs was [BMpyr][NTf_2_], and it was purchased from Iolitec (Heilbronn, Germany). Samples (40 mL) were dried and degassed under argon at 2 mbar (Heidolph rotary evaporator combined with the SC 920 G vacuum pump system) for 6 h at 378 K with an uncertainty of 1 K. Samples were stored under argon, and the water content was determined using the Karl Fischer method with a TitroLine 7500 (SI Analytics, Germany). Table 1 summarizes the specification of [BMpyr][NTf_2_].

**Table 1:** Characteristics/Specification of MWCNTs and IL Used in This Study

MWCNTs						
name	average length^3^ (μm)	average diameter^9^ (nm)	aspect ratio (−)	specific surface area (m^2^ g^–1^)	density (g cm^–3^)	carbon mass fraction purity (−)
**long MWCNTs** (in-house 16 h MWCNTs)^4^	770	60–80	11,000	22	2.1	0.98
**shortMWCNTs**^a^(Nanocyl NC7000 MWCNTs,purified)	1.5	9.5	150	300	1.75	0.90
IL						
name	acronym	CAS number	*M*	mass	water	halides
			(g mol^–1^)	fraction purity	content	(ppm)
				(−)	(ppm)	
1-butyl-1-methylpyrrolidinium bis(trifluoromethylsulfonyl)imide	[BMpyr][NTf_2_]	223437-11-4	422.41	>0.99^b^	<100^b^/46^c^	<100^b^

a Declared by the supplier (Nanocyl, Belgium); purified by subsequent washing with conc. NaOH and HCl according to ref (4).

b Declared by the supplier (Iolitec, Germany).

c Water content measured by coulometric Karl Fischer titration by a TitroLine 7500 (SI Analytics, Germany) after drying.

#### Sample Preparation

**Figure 2:**
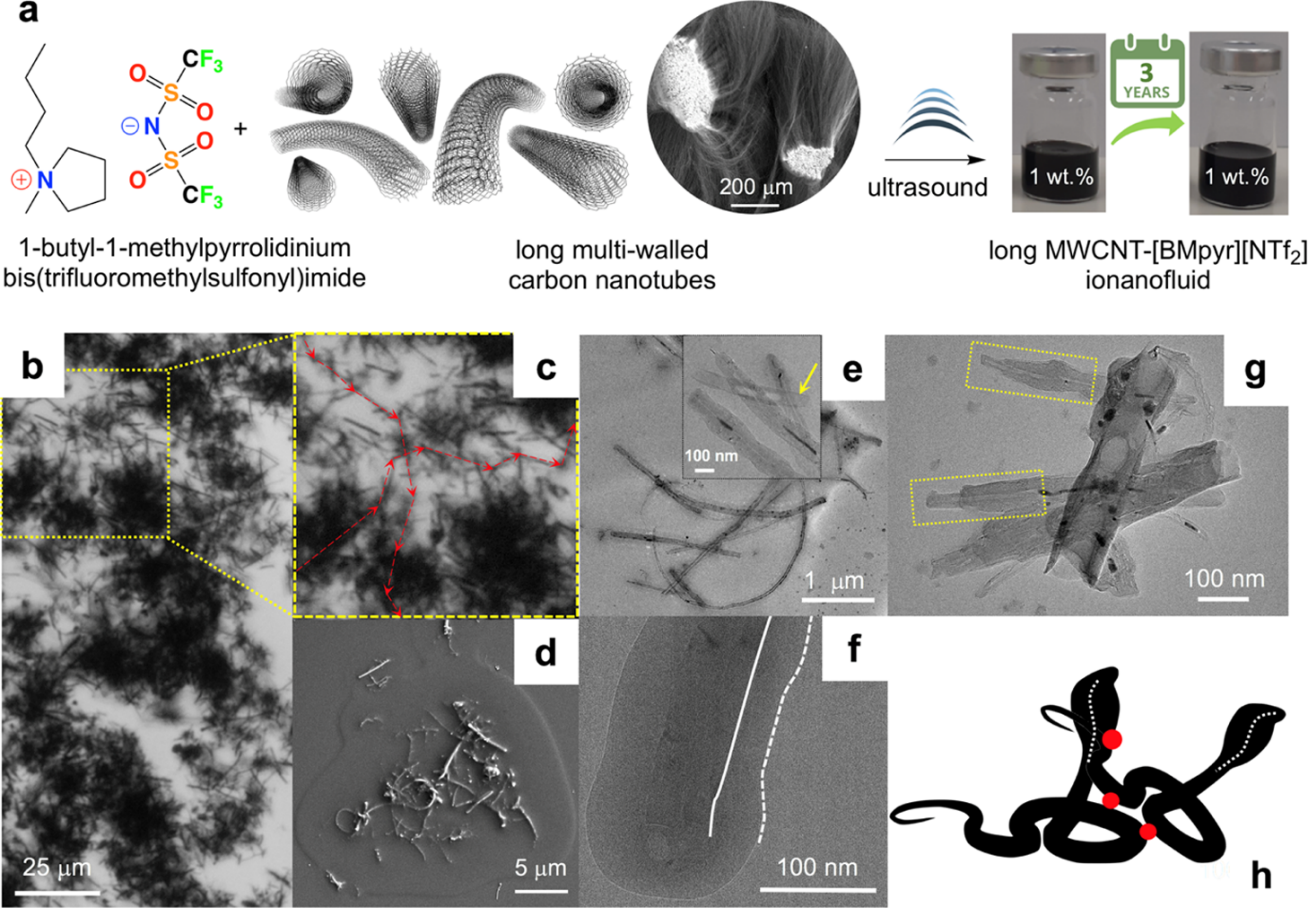
From long MWCNTs and [BMpyr][NTf_2_] to INFs. (a) Scheme of the synthesis of INFs *via* ultrasonication-assisted subzipping of the MWCNT networks; insets show a scanning electron microscopy (SEM) image of the as-grown, long, vertically aligned MWCNT bundles and vials of 3-year-stable INFs with maintained thermal conductivity. (b) Light micrograph of INFs. (c) Magnified area of MWCNT 3D networks with highlighted conduction paths. (d) Corresponding low-magnification SEM image of the INF droplet with straight and curly MWCNTs. (e) MWCNT network subzipping *via* coalescence of the nanolayer interfaces; the inset shows a single short nanotube fully unzipped into a graphene ribbon. (f) Cryo-transmission electron microscopy (cryo-TEM) image of the individual covered with the IL nanolayer, highlighting the MWCNT–INF interface. (g) MWCNT networks—with the resultant TEM image of longitudinally unzipped nanotubes (highlighted in rectangles). (h) A “cobra-like” model of subzipping of both individual and bundled MWCNTs (longitudinal subzipping; red circles represent points of contact).

INFs were prepared by a two-step procedure, that is, by dispersing powders of MWCNTs (long and short) in IL at different weight concentrations (0.2, 0.5, 0.75, and 1 wt %). Samples (20 mL) were prepared by mass using an analytical balance XA 60/220 (Radwag, Poland, with an uncertainty of ±10^–4^ g), that is, first, nanoparticles were weighed in a screw-cap bottle and then an appropriate mass of IL was added. All samples were sonicated for 10 min using a UP200St sonicator (Hielscher Ultrasonics GmbH, Germany) to apply the same protocol of preparation. During sonication, the samples were cooled by a cooling bath with ethylene glycol. The ultrasound power generator (200 W) was operated at 26 kHz frequency with 100% amplitude (nominal values). The total energy supplied to the system was 3.3 ± 0.6 kJ g^–1^. Systems with long MWCNTs (Figure 2), prepared *via* ultrasonication, formed 3-year-stable INFs at 295 and 353 K (Figure 2a). Upon ultrasonication, the as-grown long MWCNTs were cut into a length distribution with a mean of 9.5 ± 3.5 μm (min., 2.3 μm; max., 23.5 μm) (Figure 2b).

**Figure 3:**
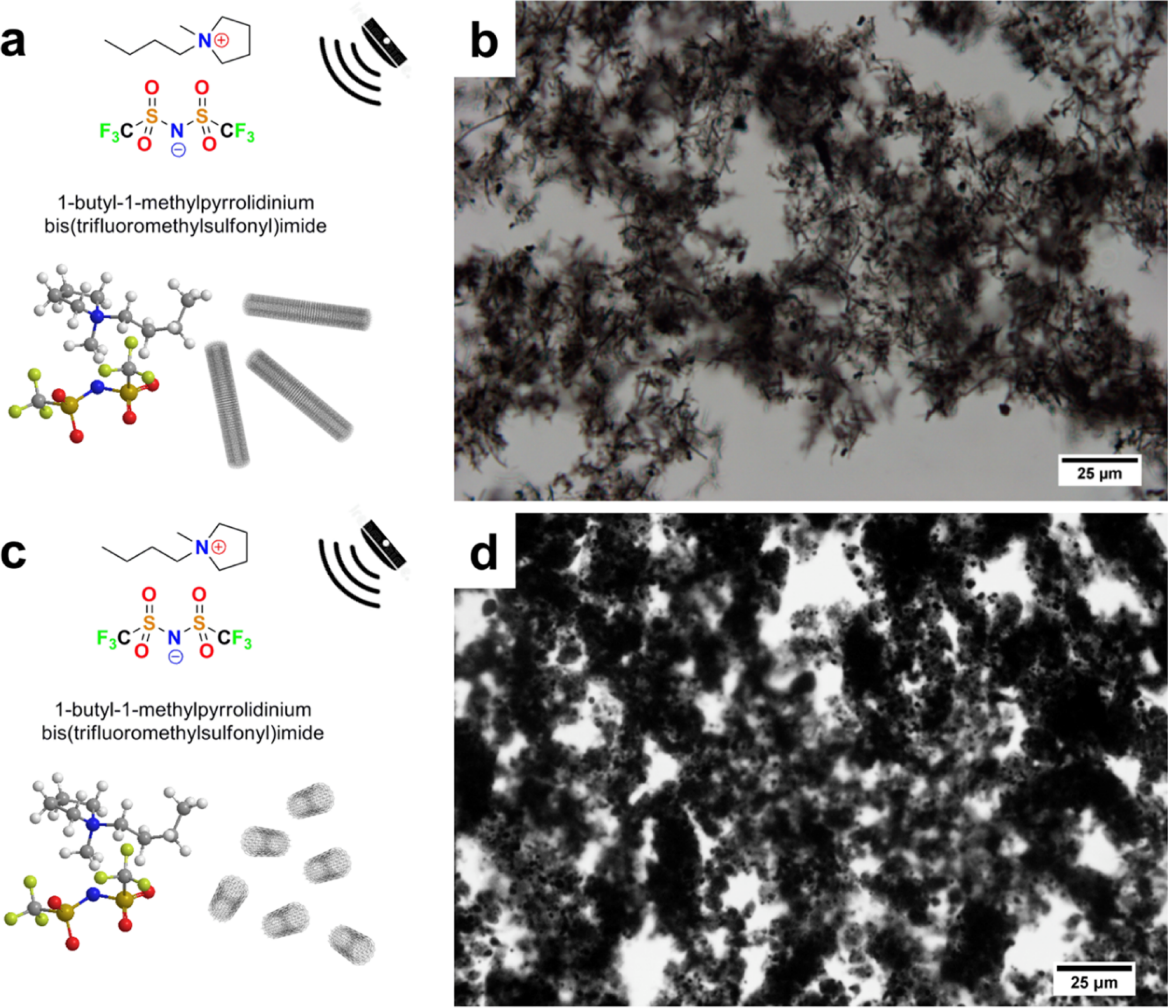
Molecular formulae of INFs: (a) INFs based on long MWCNTs and (c) INFs based on short MWCNTs. Optical micrographs of INFs: (b) INFs based on long MWCNTs and (d) INFs based on short MWCNTs.

Contrary to long MWCNTs (Figure 3a,b), the length of short MWCNTs (1.5 μm) remained significantly unchanged due to extensive entanglement of the original nanotubes into spheroidal microparticles of 2.2 μm in diameter (Figure 3c,d). INFs containing long MWCNTs yielded superstructures by long-range interactions (Figure 2c) which generated steady thermal and mechanical bridges,^10^ absent for short MWCNTs (Figure 3d).

Our developed two-step method is universal as it leads to the preparation of uniform samples in terms of the measured properties. This characteristic was verified by applying the same method to prepare the samples by independent researchers in separate laboratories at the University of Silesia in Katowice and the Silesian University of Technology. This verification was made for two INF samples of each long MWCNT composition ranging from 0.2 to 1 wt % prepared using the same batch of IL and long MWCNTs for which thermal conductivity and density were measured using the same apparatuses. Thermal conductivity is the key property characterizing the original INFs. Since density is very sensitive to any change in the INF composition, it is a great inspection tool for such purposes. Differences in thermal conductivity are in the range of 0.0 to 2.8%, while the differences in density are in the range of 0.00 to 0.03%. Thus, the reproducibility error is lower than the declared uncertainties of density and thermal conductivity measurements. It must be emphasized that analogous results were obtained when measuring the corresponding samples after they had been stored for 3 years in transparent glass vessels with a sealed cap at 295 K and exposed to daylight. Differences in thermal conductivity were in the range of 1.5 to 3.2%.

## Methods

### Optical Microscopy

Observations of the morphological structure of INFs were carried out via the conventional bright-field method using an optical microscope CH30 (Olympus, Japan) equipped with an MPlan N 50×/0.75 objective and a 5.1 MP camera ODC 832 (Kern, Germany). A 0.1 mL drop of each sample was placed between standard transparent glass microscope slides. Lengths of the MWCNT bundles were established in ImageJ software using a calibration slide provided by the manufacturer. Based on 150 measurements, the minimum value, maximum value, arithmetic mean, and standard deviation were determined.

### Scanning Electron Microscopy

SEM images were acquired using a TESCAN MIRA3 FEG-scanning electron microscope equipped with an Oxford Instruments X-maxN 80 EDS detector. Samples were sputter-coated with 10 nm of Pt using a Quorum Technologies Q150T ES sputter coater.

### Transmission Electron Microscopy

Cryo-TEM micrographs were obtained using a Thermo Scientific (FEI Company) Talos F200X G2 microscope operating at 200 kV. Images were recorded at a low dose using a Ceta 4k × 4k CMOS camera and acquired through Velox software. Specimens were vitrified by plunge-freezing an aqueous suspension of CNTs in an ionic liquid onto copper grids (300 mesh) with a lacey carbon film. Prior to use, the TEM grids were glow-discharged for 60 s at a current of 25 mA using a Quorum Technologies GloQube instrument. A suspension of the sample (2.3 μL) was pipetted onto the TEM grid, blotted for 3 s at blot force −5 using dedicated filter paper, and immediately frozen by plunging into liquid ethane utilizing a fully automated and environmentally controlled blotting device, Vitrobot Mark IV. The Vitrobot chamber was set to 277.15 K and 95% humidity. After vitrification, the specimens were kept in liquid nitrogen until they were inserted into a Gatan Elsa cryo-holder and imaged by TEM at 95.15 K. Room-temperature (293 K) TEM samples were prepared by pipetting 2 μL of CNTs in an IL onto a copper grid (300 mesh) with a continuous carbon film followed by blotting to remove the excess IL.

### Raman Experiment

A WITec confocal alpha 300R Raman microscope (CRM) was used herein. The Raman experiment was performed at 77 K using the Linkam THMS600 stage. Samples were cooled with a cooling rate of 50 K min^–1^, and the uncertainty of temperature measurements was 1 K. The experiment was performed using a solid-state laser operating at 532 nm (15 mW at the sample) coupled to a confocal microscope *via* a single-mode optical fiber with a diameter of 50 μm. Incident and scattered laser radiation were passed through an Olympus MPLAN 50×/0.76NA air objective. The scattered line was focused onto a multimode fiber (50 μm diameter) and a monochromator. The spectrometer monochromator was calibrated using the emission lines of a Ne lamp, while the signal of a silicon plate (520.7 cm^–1^) was provided for checking the beam alignment. For each sample, 10 spectra were recorded, while each of them was measured using 20 accumulations, with integration times of 10 s and a resolution of 3 cm^–1^. Postprocessing analysis, including cosmic ray removal, baseline correction, and spectrum averaging for the individual sample, was performed using WITecProjectFive Plus software. Finally, the averaged spectra were subjected to Gaussian–Lorentz band fitting analysis using the Grams 9.2 software package to estimate the absolute position, intensity, integrated intensity (=area), and full width at half maximum (FWHM) of the bands related to the IL and CNTs.

### Thermal Conductivity Measurements

The thermal conductivity of INFs at 298.15 K was measured in triplicate and averaged using a KD2 Pro Thermal Properties Analyzer (Decagon Devices Inc., USA) with a single needle KS-1 sensor that is 1.3 mm in diameter and 60 mm in length. KD2 Pro works based on the hotwire technique in which a thin (electrically insulated) conducting wire immersed in the test medium is used as the line heat source and temperature sensor. The KS-1 needle generates a very small amount of heat to the sample during measurement, minimizing problems with free convection. The estimated expanded uncertainty (*k* = 1, 95% confidence level) of the thermal conductivity measurements was estimated to be ±5%. The uncertainty of temperature measurements was 0.05 K.

### Rheological Measurements

Rheological properties of the base IL and INFs (samples of 20 mL) were tested using a rheometer MCR 302 (Anton Paar, Austria) with a cone-plate geometry system CP50-1° (50 mm diameter, 1° cone angle, 0.1 mm gap width, smooth). Measurements at 298.15 K included the determination of flow/viscosity curves (shear rate from 0.1 to 100 s^–1^), hysteresis loops (interval I from 5 to 131 s^–1^ for 300 s, interval II at 131 s^–1^ for 180 s, and interval III from 131 to 5 s^–1^ for 300 s), and storage and loss moduli *G*′ and G″ (strain sweep from 0.01 to 100%, with a constant oscillation frequency of 1.59 Hz = 10 rad s^–1^). The temperature was maintained by the Peltier temperature control unit with fluctuations not exceeding ±0.01 K. The uncertainty of temperature measurements was 0.02 K. The uncertainty of torque measurements was ±0.05 μNm.

### MD Simulations

Simulations were used to investigate the effect of the distance between two CNTs on the organization and conformation of the IL ions. Since the CNTs studied in this work have diameters > 9.5 nm, it was considered that the nanotube curvature could be ignored and therefore the carbon surfaces (CSs) could be modeled as graphene surfaces. For this, two sheets with 528 carbon atoms forming a hexagonal grid with bonds of 0.1421 nm length were placed at different distances (1.0, 1.5, 2.0, 3.0, and 4.0 nm), parallel to the *XY* plane, in a simulation box with 3.96 nm × 3.86 nm × 10.0 nm. A total of 250 IL ion pairs were then randomly distributed inside the box, ensuring that the same number of cations and anions were placed between and outside the CS. Periodic boundary conditions were considered along all dimensions, but box size changes were only allowed along the *Z*-axis. This allowed us to model the CS as “infinite” structures along the *XY* plane. Equilibration of the simulation boxes was achieved by performing several MD runs of 1 ns until a constant volume and system energy was obtained. The final production stage consisted of 2 ns simulation runs, where the trajectory was recorded every 1 ps. A timestep of 2 fs was used in all stages of the simulations with a cutoff distance of 1.5 nm for both van der Waals and Coulomb interactions. The Ewald summation technique was employed to account for the electrostatic interactions beyond the cutoff distance. The Nosé–Hoover thermostat and barostat were used to control the temperature at 298.15 K and pressure at 0.1 MPa. Details and an example of the simulation boxes obtained after the simulations are given in Table S1 and Figure S1 in Supporting Information. The force field used in the simulations was based on the following parametrizations: (i) the interactions between the IL ions were computed as in the CL&P model;^11,12^ (ii) the interaction between the cations and anions with the carbon surface was retrieved from the parametrization previously reported by França and coworkers;^13^ and (iii) the CSs were simulated using the OPLS-AA model, which was previously found suitable for this type of simulation.^13,14^ Simulations were prepared using Packmol^15^ and DLPGEN.^16^ All MD calculations were performed with LAMMPS.^17^ The interaction between CSs and the IL was investigated using the program AGGREGATES,^18^ and the details are given as the Supporting Information.

## Results and Discussion

### Discovery of “Cobra-like” CNT Networks in the IL

**Figure 4:**
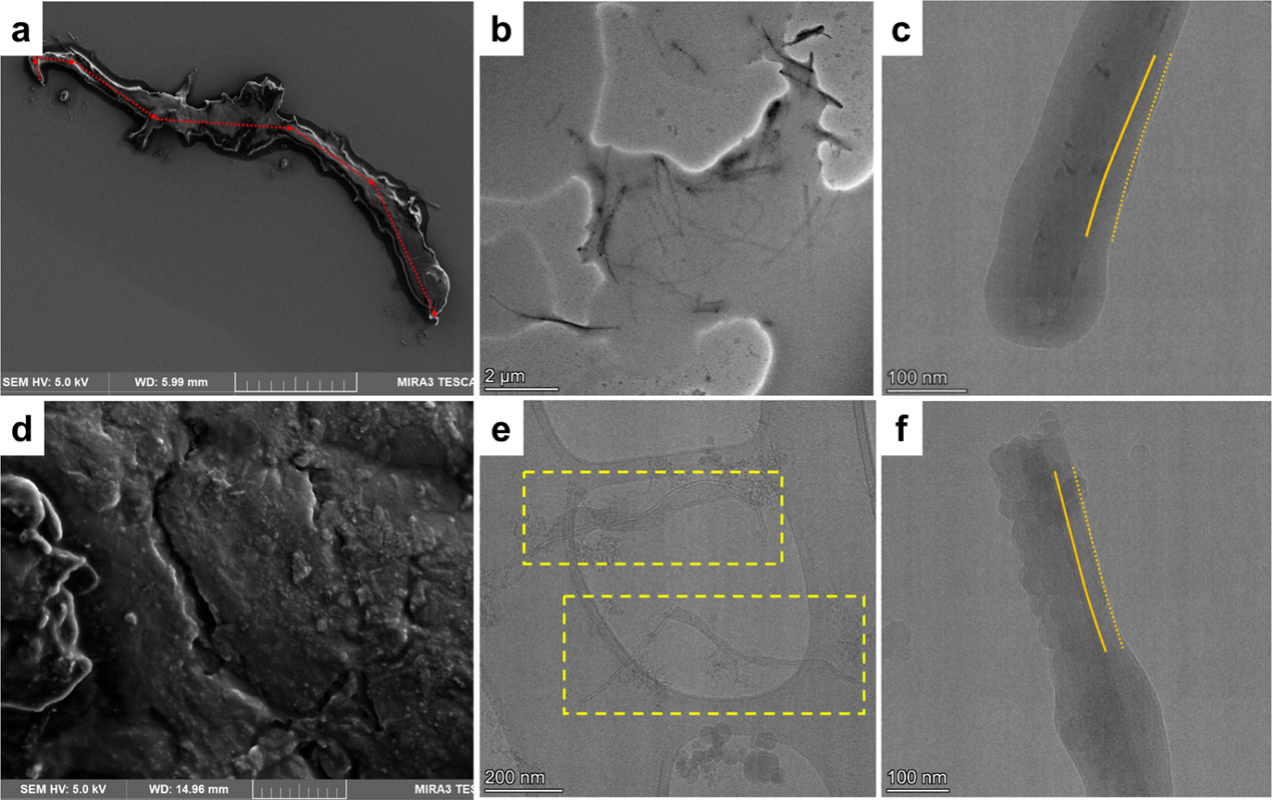
SEM and cryo-TEM images of INF. (a) INF droplet composed of long MWCNTs. (b) Cryo-TEM image revealing nanotube subzipping via coalescence of the nanolayer interfaces for long MWCNTs. (c) Cryo-TEM image revealing the thickness of the interface nanolayer (as demarcated with solid and dashed lines) of 25 ± 11 nm for long MWCNTs. (d) INF droplet composed of short MWCNTs; scale is 5μm (e) Cryo-TEM images revealing mostly separated nanotubes, zipped, and locally unzipped *via* coalescence of the nanolayer interfaces. The outermost nanotube walls additionally emerged as incompletely exfoliated; the highlighted areas in (e) show the zipped and partially unzipped short bundles of individual nanotubes toward limited functionality. (f) Cryo-TEM image revealing the thickness of the interface nanolayer (as demarcated with solid and dashed lines) of 27 ± 12 nm for short MWCNTs.

How the long MWCNT-based superstructure, in the background of short MWCNTs, is organized at the molecular level has become an urgent question. To address this question, we performed cryo-TEM studies to track the interfacial (IF) nanolayer thickness and the way how the nanotubes interconnect *via* the nanolayers (Figure 4). Initial SEM screening showed long MWCNT-based INFs as exceptionally fibrous, highly ordered droplets (Figure 4a) opposite to more isotropically distributed short MWCNTs (Figure 4d).

As the cryo-TEM imaging revealed, in all of the INFs with long MWCNTs, subzipped nanotube systems of lengths from several hundreds of nanometers to a few microns were found (Figure 4b) with some examples of exfoliation of the most outer walls due to the IL-assisted ultrasonication. Thus, subzipping is a result of the intertube zipping together with locally unzipped individual pairs of nanotubes and/or longitudinally unzipped nanotubes. The formation of 3D networks leads to enhanced stability of high-performance INFs. As confirmed by optical microscopy, INFs yielded superstructures by long-range interactions (Figures 2c and also 3b). MWCNTs formed compact networks composed of straight and bent nanotubes toward the interconnected superlattice vibration pathways (Figure 2d). Therein, the physical intertube contact could generate steady thermal and mechanical bridges.^10^ TEM revealed partially subzipped nanotube systems of lengths from subnanometers to a few microns (Figure 2e)—with examples of exfoliation of the outermost walls *via* IL-assisted ultrasonication. This phenomenon was confirmed by cryo-TEM (Figure 4b). The nanotube system represents a “cobra-like” model (Figure 2g,h) of subzipping both individual MWCNTs and the MWCNT networks (Figure 2g).

Opposite to long MWCNTs, short MWCNTs—though coalescing up to a few nanotubes—were unable to form subzipped nanotube bundles into the functional networks (Figure 4e). Separated individual pairs of zipped and locally unzipped short nanotubes can be observed (Figure 4e). Notably, the individual nanotubes were covered with from a few to several nanometer-thick IL layers. The thickness of the interface nanolayer on a separate nanotube was practically independent of the nanotube type, that is, 25 ± 11 nm for long (Figures 2f and 4c) and 27 ± 12 nm for short (Figure 4f). The observed shape of the nanolayers was from uniformly ovoid (Figure 4c) to multicentered (Figure 4f). Overall, the phenomenon of CNT networking would suggest the coalescence of nanotubes driven by the elastic IL interfaces at the junctions. Moreover, while the nanotube coalescence phenomenon is well known in the formation and processing of CNT fibers (*per analogiam* to nematic phase in liquid crystals),^19,20^ observation of the sole IL nanolayers independent of the nanotube morphology and surface chemistry has not been reported to date.

### IL-Stabilized Subzipped CNT Networks

**Figure 5:**
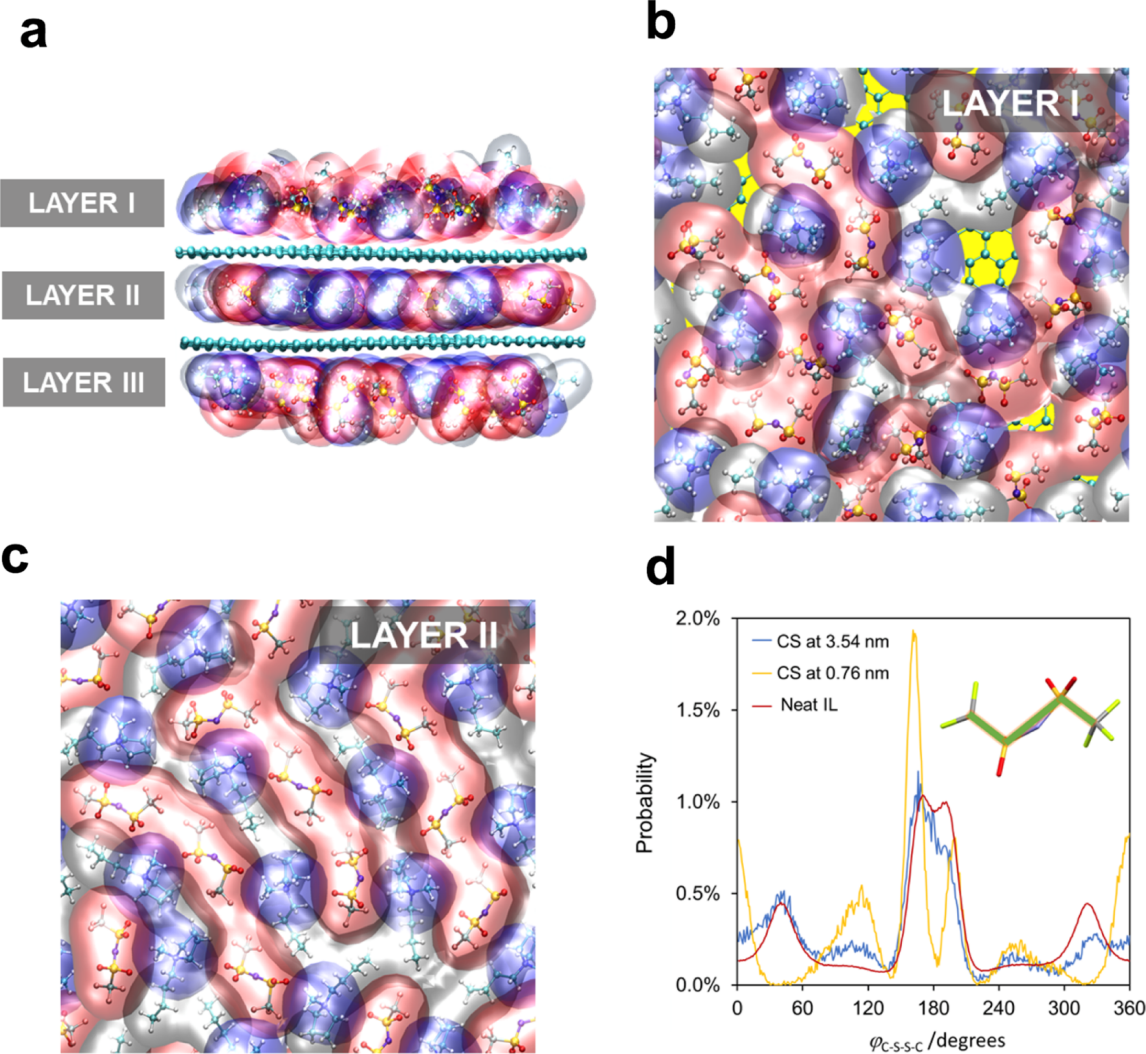
MD simulations of the CNT–IL interfaces. (a) Side views of IL ions in contact with two CSs (green) placed at 0.76 nm distance from each other. (b,c) Top views of IL ions. (a–c) Volume occupied by the molecules, computed based on the atoms van der Waals radii,^21^ colored as follows: positive charge and alkyl chain of cations in blue and gray, respectively, and anions in red. (d) Comparison of the dihedral angle distribution, defined between carbon and sulfur atoms of [NTf_2_]^−^ (inset), in neat IL (red) and located in layer II at CS distances of 3.54 nm (blue) and 0.76 nm (yellow).

To gain further insight into the nanoarchitecture of INFs in MD simulations (Figure 5), three layers of ions were defined (Figure 5a): layers I and III, in which the ions face the bulk dispersion, and layer II, where the ions are located between the two carbon sheets (CSs) mimicking the outermost CNT walls. The organization of molecules in layers I and III was found to be similar. When the number of IL pairs between CSs was such that only one layer of ions was formed, then the ions produced a cohesive and ordered structure (Figure 5c). In turn, when the IL layer faced the liquid bulk, a loose structure was formed, and the molecules could easily swap positions near CS (Figure 5b). This dynamic also led to the momentaneous formation of “holes” in this layer (yellow areas in Figure 5b). In the central layer, the molecules are aligned parallel to the surface, suggesting that they are restrained to in-plane movements, while in layers I and III the molecules exhibit translational and rotational degrees of freedom (Figure 5a). Therefore, the cohesive nature of layer II located between the two CSs (which rapidly vanishes with the separation of the CSs) leads to a more efficient and stable charge balance of the ions close to the surface. As a result, the obtained structure is more stable than the arrangements in which the two CSs are separated (Table S1 and Figure S1 in the Supporting Information).

When two adjacent CNTs, approximated by CSs, were separated by a distance *d*_CS_ > 2 nm, the obtained interaction energy with the IL was approximately *U*_int_ = −34 kJ mol^–1^ (Figure S1). This absolute value slightly increases as this distance is reduced until one IL layer between the walls of adjacent CNTs is formed (*d*_CS_ = 0.76 nm). At this point, *U*_int_ = −38.2 kJ mol^–1^, which corresponds to an internal energy variation of Δ*U* = −4.2 kJ mol^–1^. Hence, this result suggests a negative Gibbs energy variation for the formation of a structure where adjacent CNTs are separated by one layer of IL ions, relative to one where the CNTs are separated by a long distance (considering that this process is essentially controlled by Δ*U*). Thus, the simulation data propose that the formation of the structure shown in Figure 5a is favorable from a thermodynamic point of view.

### CNT–IL Interface—the CNT Perspective

**Figure 6:**
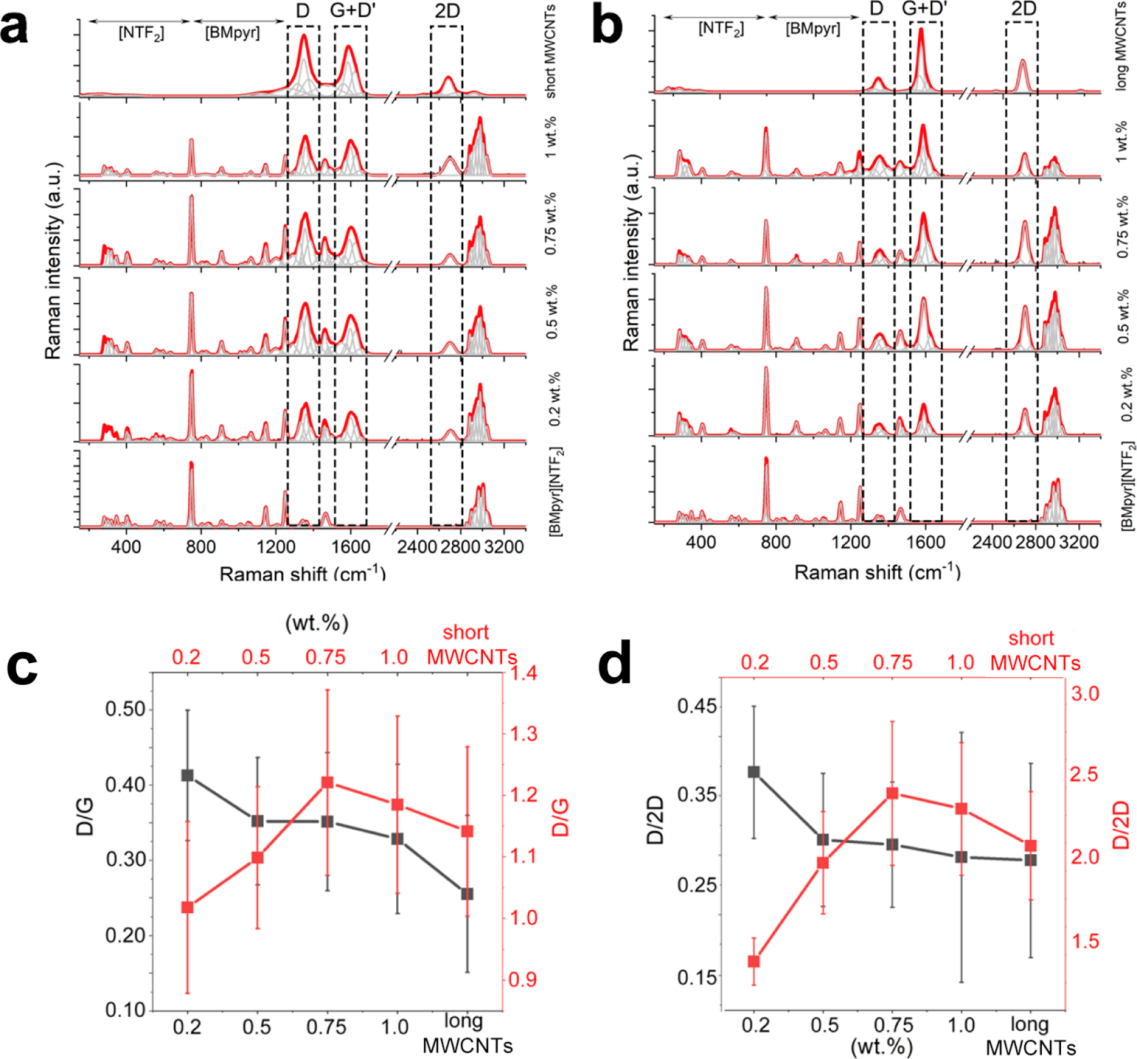
Raman spectra of the IL, INFs, and MWCNTs. (a) INFs with reference to short defective MWCNTs. (b) INFs with long crystalline MWCNTs. (c) Integral intensity ratios of D/G. (d) Integral intensity ratios of D/2D.

Studying the character of MWCNT–IL interactions *via* Raman spectroscopy from the “CNT perspective” (Figure 6), we encountered an upshift of the prominent Raman bands as a function of the MWCNT loading (Figure 6a,b). This effect correlates with the bundle-penetrating power by IL weakening tube–tube interactions *via* subzipping of the long MWCNT networks.^22^ Importantly, we see that the *in situ* formation of INFs bears distinct hallmarks of the *synthesis*. Upon ultrasonication-supported cutting of long MWCNTs, the nanotubes are quenched *via* covalent bonds with the involvement of cations. The “new” MWCNTs subzip and become tip-/edge-functionalized, which enhances their dispersibility and hence functionality. Moreover, interactions between the individual nanotubes partially change from π–π stacking into more complex interactions *via* functionalized “contacts”, enabling subzipping of the CNT network. Such modifications are in line with the macroscale properties of INFs, as the contacts between rigid and flexible (local functionalization induces bending), geometry-driven long MWCNT superlattices are scaffolded by CNT proximity-stabilized [NTf_2_]^−^ conformations.

The G-, D-, and 2D-bands stand for the (i) graphitic in-plane bond stretching mode of the C–C bonds in the hexagonal lattice; (ii) defect-sensitive, for example, sp^3^-carbon-related, first-order component of the hexagonal-breathing mode derived from an elastic scattering of a photoexcited electron by the defect; and (iii) another defect-sensitive scattering band originating from the vibrational breathing mode of the six atoms of the hexagon). However, more detailed information provided the analysis of the integrated intensity ratios *I*_D_/*I*_G_ and *I*_D_/*I*_2D_. In our case, *I*_D_/*I*_G_ and *I*_D_/*I*_2D._ for long MWCNTs decreased with concentration (Figure 6c,d), which confirmed higher functionalization levels accompanying the subzipping of the individual nanotubes into the edge-modified graphene ribbons. This tendency—at lower concentrations—was opposite for short, more defective MWCNTs since they were richer in the more reactive sp^3^-carbon atoms. In this case, the net effect of “nanotube cleaning” *via* exfoliation toward soluble species was observed. For both MWCNT types, new covalent bonds between the wall and the IL cation were formed. However, sp^3^-carbon atoms were more reactive than sp^2^-aromatic atoms in the more graphitized, smooth MWCNTs (Figure 6c,d). Such a “cation capture” would also neutralize dangling bonds upon MWCNT ultrasonication-induced cutting, possibly *via in situ* methylation and expulsion of the tertiary amine.^23^ Both the cutting effectiveness and the intensity of functionalization decreased with the INF viscosity, suggesting a reaction under the diffusion regime. Importantly, the tendencies in the IL-induced MWCNT modifications were dissimilar for short and long MWCNTs. For short MWCNTs, originally richer in sp^3^-defects, at lower MWCNT concentrations, exfoliation of the less tightly bound outer walls dominated. This behavior first exposed the more graphitized, deeper walls. Moving to the higher short MWCNT concentrations, we encounter the *I*_D_/*I*_G_ maximum related to the generation of the most abundant novel defects by neutralization of free radicals upon the outer wall exfoliation. In turn, the “cleaning” effect and the overall reactivity were inhibited by the high INF viscosity suppressing the possible cutting and so the reactions of dangling bonds. Whereas for long MWCNTs—displaying the more aromatic sp^2^-character (hence rigidity and geometry-driven self-assembling), higher level of graphitization, enhanced smoothness, and a high aspect ratio—the stage of exfoliation was negligible, and a decreasing tendency in (i) cutting and (ii) functionalization levels was found. Both the cutting effectiveness and functionalization degree decreased with the INF viscosity. A slightly lower upshift of all Raman bands for the long MWCNTs resulted from subzipping. In total, long MWCNTs were less “debundled”, whereas simultaneously, short, sp^3^-defectuous MWCNTs emerged as more strongly interacting—in all aspects—with the IL molecules. Concerning the CNT–IL interface from the molecular point of view, and since all G- and D-bands from long MWCNTs in INFs were shifted to higher frequencies, one might assume the π-cation interactions and hence the electron-withdrawing effect of cations.^24^

### CNT–IL Interface—the IL Perspective

**Figure 7:**
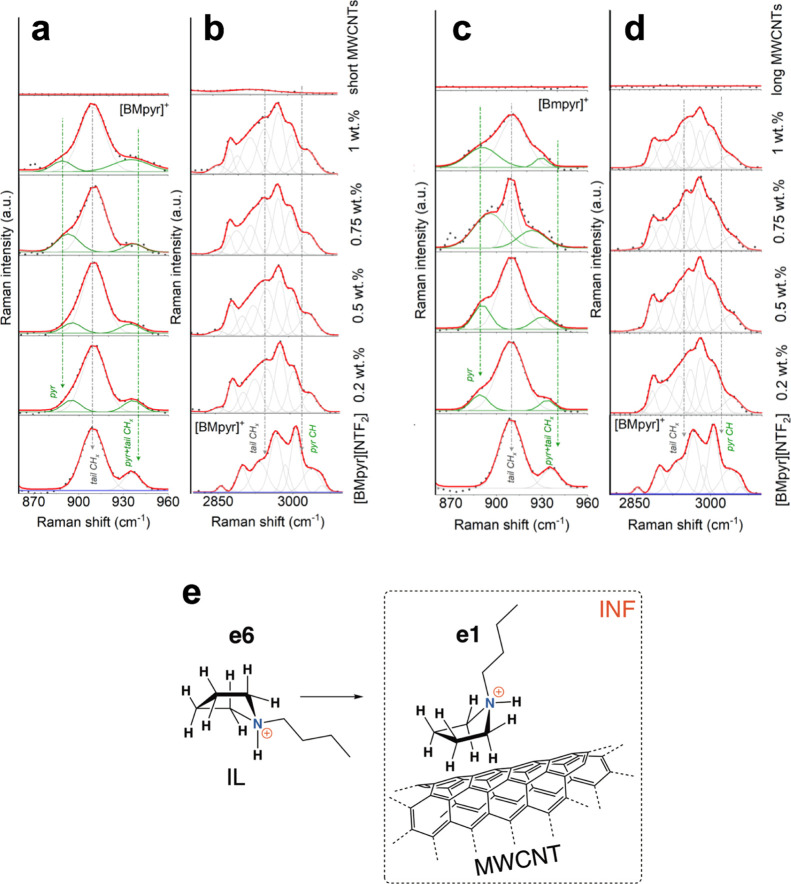
Raman spectra of the reference IL and INFs presented in various ranges. (a,b) Raman spectra of the reference IL and INFs with increasing amounts of short MWCNTs. (c,d) Raman spectra of the reference IL and INFs with increasing amounts of long MWCNTs. (a,c) presents the range of 960–860 cm^–1^ ([BMpyr]^+^), and (b,d) presents the range of 3200–2800 cm^–1^ ([BMpyr]^+^). Main bands that indicate structural changes are color- and text-marked. Band fitting analysis was performed in the Grams 9.2 software package to look into more detail on the nature of structural alterations. (e) More ordered MWCNT walls enforce the e6 → e1 [BMpyr]^+^ isomerization.

Similarly, interesting outcomes were found from the IL structure perspective, both for neat ILs and INFs by Raman spectra (Figure 7). In the fingerprint regions of the IL in INFs, strong band reorganization at approximately 3200–3000 cm^–1^ and a slight modification in the 3000–2800 cm^–1^ region suggest structural changes within the pyrrolidinium and insignificant alteration of the *n*-butyl tail in INFs, respectively (Figure 7b,d).

The other region, that is, 1100–800 cm^–1^ turned out to be conformation-sensitive (considering the above moieties) and proved the presence of only two unique Raman modes at 934 and 909 cm^–1^ for the reference IL spectrum (Figure 7a,c) corresponding to the e6 eq-envelope cation conformers.^25^ Nevertheless, both MWCNT-based INFs manifested the additional band located at a lower frequency that corresponds to the e1 ax-envelope cation isomer with the *n*-butyl chain in the axial position relative to the ring plane.^25^ Notably, the mutual intensity between the e1- and e6-bands was independent of the MWCNT content for short MWCNT-based INFs (Figure 7a), while it was found to be increasing for the long MWCNT-based INFs (Figure 7c). Hence, the more ordered, aromatic, and rigid CNT walls enforce the e6 → e1 isomerization, which becomes particularly favorable in the case of a considerable area of well-developed graphene walls in INFs (Figure 7e).

**Figure 8:**
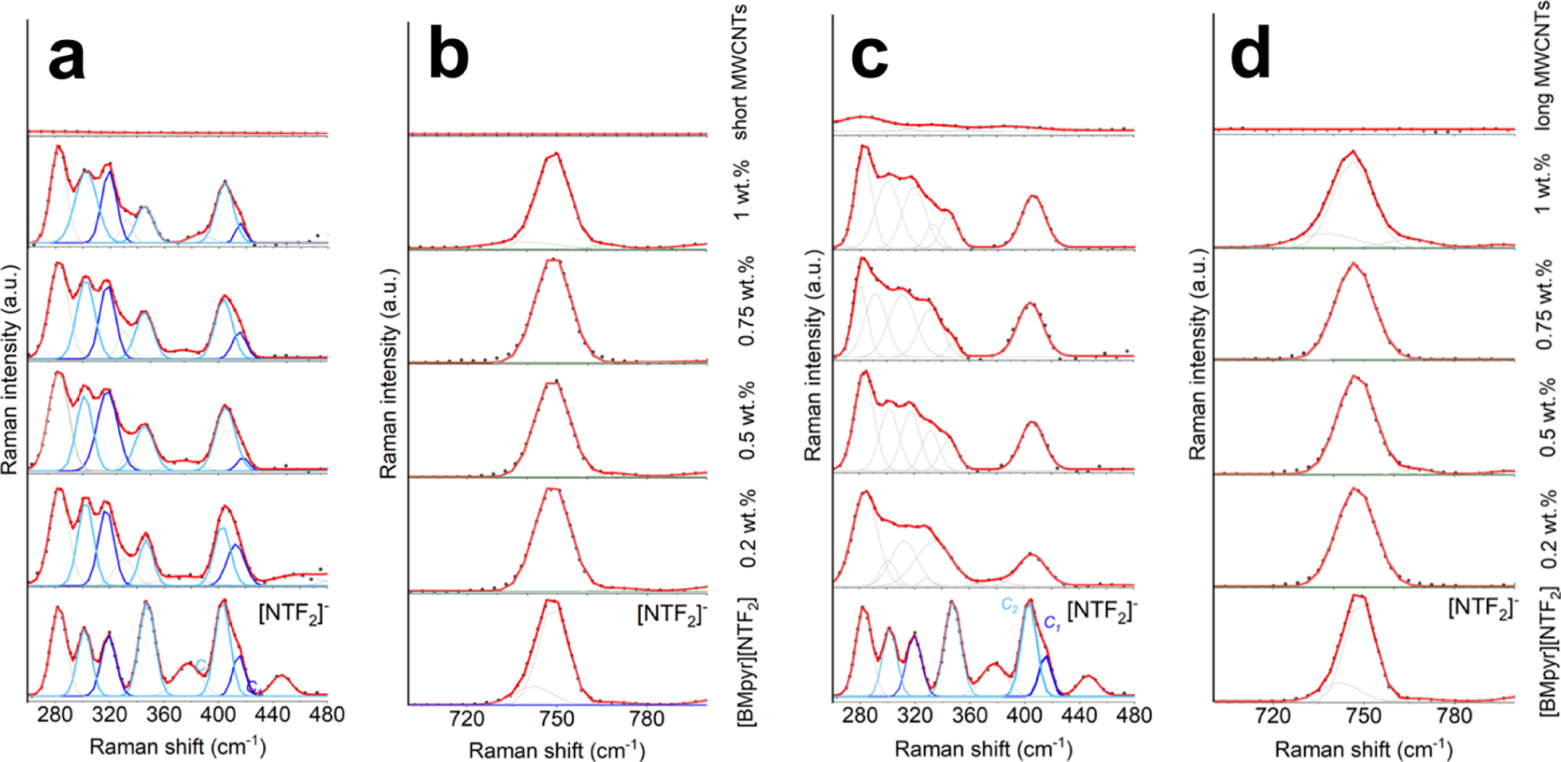
Raman spectra of the reference IL and INFs presented in various ranges. (a,b) Raman spectra of the reference IL and INFs with increasing amounts of short MWCNTs. (c,d) Raman spectra of the reference IL and INFs with increasing amounts of long MWCNTs. (a,c) presents the range of 480–260 cm^–1^ ([NTf_2_]^−^), while (b,d) presents the range of 800–700 cm^–1^ ([NTf_2_]^−^). Main bands that indicate structural changes are color- and text-marked. Band fitting analysis was performed in the Grams 9.2 software package to look into more detail on the nature of structural alterations.

Further, the 800–700 and 480–260 cm^–1^ regions inform about the [NTf_2_]^−^ anion structure and its interactions with the environment (Figure 8), and they were extensively studied for the neat IL.^26−31^ Analysis of these fingerprint regions proved the *trans* conformation (C2) of the [NTf_2_]^−^ anion as the dominating one (with a limited number of “free” *cis* conformation C1).

This effect was found actually for the neat IL and short MWCNT-based INFs regardless of the CNT content (Figure 8a). INFs with long MWCNTs tended, in a more pronounced manner, to equalize the population of *trans* conformers (C2) and *cis* conformers (C1) of [NTf_2_]^−^ by hampering the free rotation in the cation conformation by its complexation caused by its *in situ* covalent functionalization with the CNT surface. This reflects the increase in the intensity of bands in the 340–260 cm^–1^ range compared to the 480–340 cm^–1^ region (Figure 8c).

Referring back to MD simulations to verify if the tight molecular organization observed in layer II can be related to the experimentally observed Raman results, the conformations of the anions in contact with the carbon surfaces and in neat IL were investigated. For this, the probability distribution of the dihedral angle formed between the carbon and sulfur atoms of [NTf_2_]^−^ was evaluated (φ_C–S–S–C_; see inset Figure 5d). From this analysis, if φ_C–S–S–C_ ≈ 0° or φ_C–S–S–C_ ≈ 360°, the molecules are in a *cis* conformation (C1). In turn, angles close to φ_C–S–S–C_ ≈ 180° imply a *trans* conformation (C2). If CSs were far apart, a small difference between the distribution of φ_C–S–S–C_ existed relative to that in neat IL (Figure 5d). Additionally, the distribution of the angles was consistent with the majority of the molecules being in the C2 conformation (φ_C–S–S–C_ ≈ 180°). If CSs became closer, and their distance was compatible with the formation of one layer of ions between CNTs (*d* = 0.76 nm), a significant increase in the probability of finding anions with φ_C–S–S–C_ ≈ 0° or φ_C–S–S–C_ ≈ 360° was noticed (C1 conformation) in layer II. Then, the cohesive nature of layer II, imposed by the close CSs, favors the enrichment of the C1 conformer of [NTf_2_]^−^ in INF. A similar analysis performed on the [BMpyr]^+^ alkyl chain revealed no conformational change with the distance between CSs (Figure S2 in the Supporting Information).

### High Thermal and Rheological Performances

**Figure 9:**
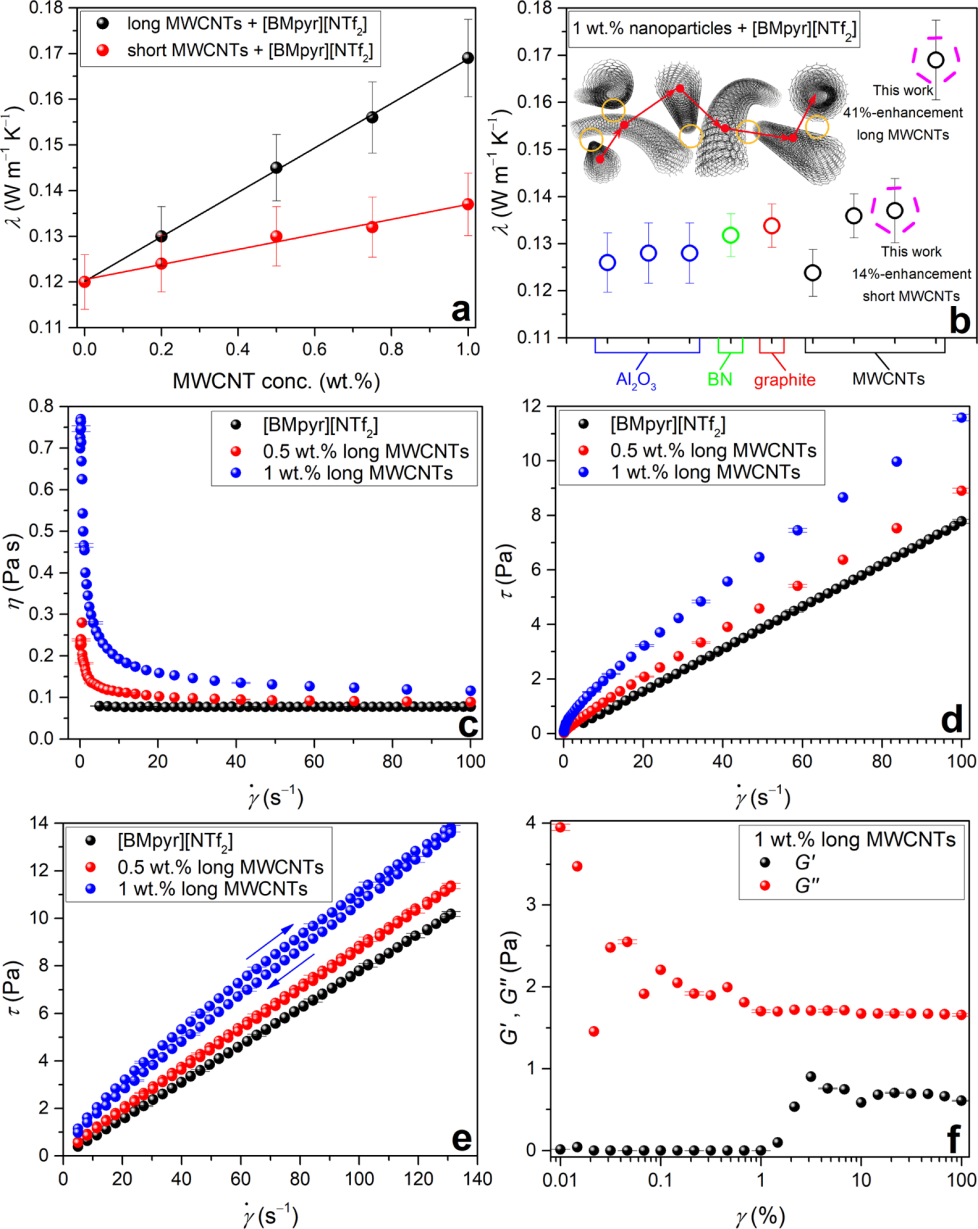
Thermal conductivity and rheological properties of [BMpyr][NTf_2_]-based INFs. (a) Thermal conductivity λ at 298.15 K for INFs containing long or short MWCNTs. Data represent the average values of technical triplicates (see Table S2). Solid lines represent simple regression lines on the confidence level of 95% (*r*^2^ = 1.00 and *r*^2^ = 0.99 for INFs with long and short MWCNTs, respectively) and by two-sided *t*-test. (b) Record-breaking λ of long MWCNT-based INFs and the literature data.^32−35^ (c–f) Rheological characteristics of long MWCNT-based INFs at 298.15 K, which include viscosity curves (c), flow curves (d), hysteresis loops (e), and storage and loss moduli (f) where γ̇ is the shear rate, γ is the shear strain, η is the viscosity, τ is the shear stress, *G′* is the storage modulus, and *G″* is the loss modulus. Data represent the results for distinct samples. Error bars represent measurement uncertainties.

The most critical parameter in the figure-of-merit for INFs is thermal conductivity. Here, for the first time, we observe that the INF composed of 1 wt % long MWCNTs + [BMpyr][NTf_2_] displays a major enhancement of thermal conductivity, that is, 41%. The perfection of the MWCNT nanoarchitecture as a thermoactive component with its supramolecular arrangement and MWCNT–IL interactions must transfer to the record-breaking thermal conductivity of INFs based on long MWCNTs and [BMpyr][NTf_2_] against the literature data^32−35^ (see Figure 9a,b and also Table S2 in the Supporting Information). Indeed, such phenomenon is derived from the optimally subzipped MWCNT 3D network at the nanotube’s given aspect ratio and the graphitization level. These factors allow individualization of the originally sp^2^-crystalline MWCNTs and induce changes toward the local alignment of IL ions in their more stable conformations. Such individualization is additionally supported by the covalent tip-modifications of MWCNTs upon their ultrasonication-induced breaking/cutting. In turn, modification of [BMpyr][NTf_2_] by 1 wt % short MWCNTs resulted in an increase of thermal conductivity by 14% (Table S2), which is thrice and one-and-half times higher than that recorded by Nieto de Castro *et al.*(32) and Oster *et al.*,^33^ respectively, with different MWCNTs.

**Figure 10:**
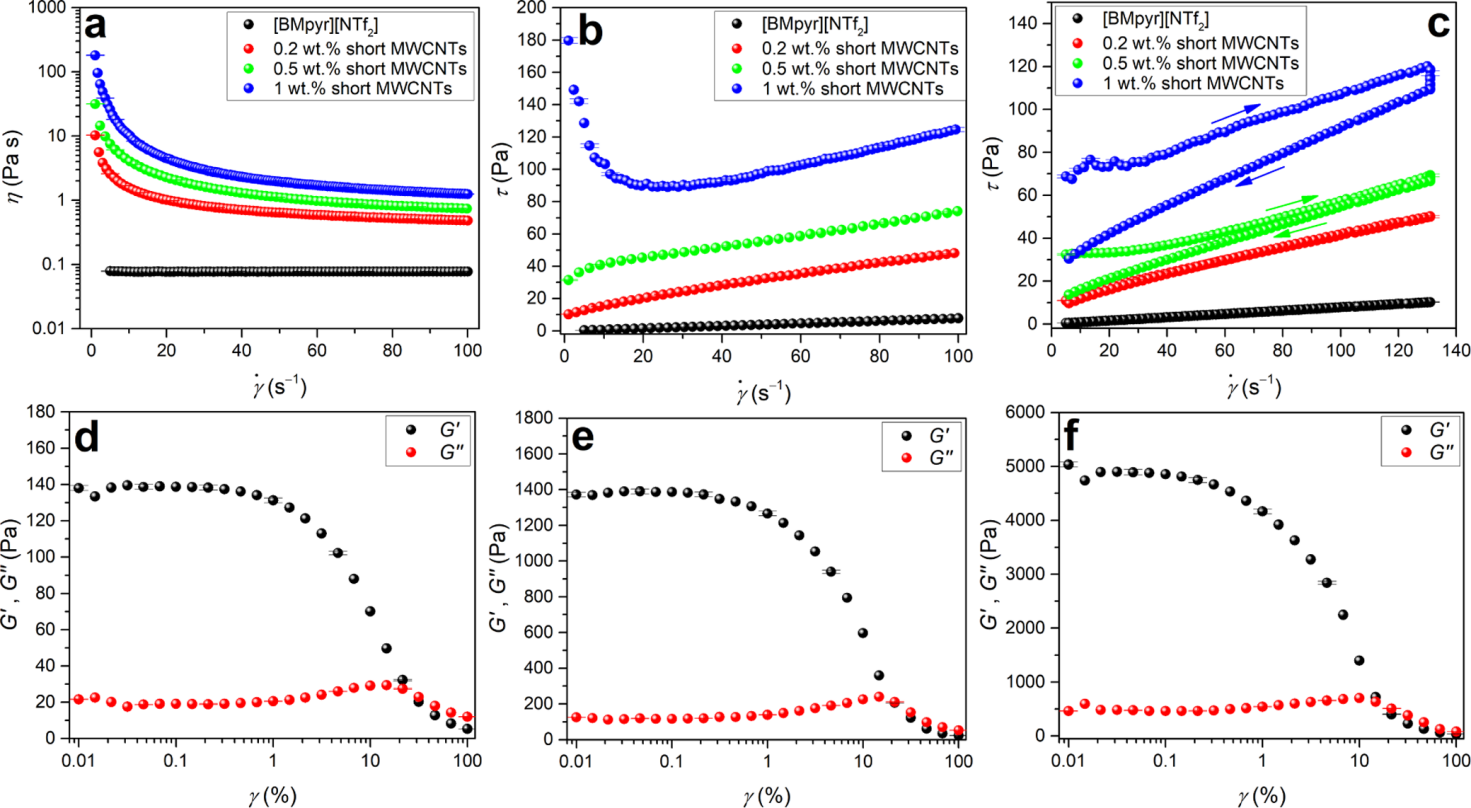
Rheological characteristics at 298.15 K of INFs containing short, poorly crystalline MWCNTs and [BMpyr][NTf_2_]. (a) Viscosity curves. (b) Flow curves. (c) Hysteresis loops (obtained at the same time program, see Experimental Section). (d–f) Storage and loss moduli (*G*′, *G*″) of INFs with 0.2, 0.5, and 1 wt % short MWCNTs, respectively. Error bars represent measurement uncertainties.

For heat-transfer fluids, apart from time and operational stability, the enhanced thermal conductivity should be supported by the optimal internal friction during flow. Thus, the key challenge in the design and synthesis of INFs that shall fulfill the most stringent criteria for heat-transfer fluids is their minimized viscosity to avoid high-energy consumption upon pumping. It is therefore indeed very prospective that the INFs based on long, crystalline MWCNTs enabled the formation of INFs of more than 2 orders of magnitude lower viscosity than their IL-thickening C-sp^3^-rich counterparts (compare Figures 9c and 10a). Principally, [BMpyr][NTf_2_]-based INFs with either MWCNTs are non-Newtonian shear-thinning, that is pseudoplastic media (Figures 9c,d and 10a,b). Both the viscosity and non-Newtonian properties of INFs increased with the MWCNT concentration due to the formation of larger networks and their reversible though intensified subzipping with increasing shear rate, respectively. However, the 1 wt % long-MWCNT INF displays insignificant thixotropic properties with a small hysteresis loop area (Figure 9e). Finally, INFs turned out to be viscous liquid-like media with negligible elastic properties (Figure 9f), which indicates their high performance.

For INFs composed of 1 wt % short MWCNTs of high surface area, the yield stress appeared, that is, the minimum shear stress required to initiate flow (Figure 10b). It means that the INFs with such nanotube loadings were in fact nonlinear viscoplastic fluids. Further research revealed that INFs with high loadings of those short, defective MWCNTs (≥0.5 wt %) had a memory of deformation history, also referred to as thixotropy (Figure 10c). The structure of these INFs was broken down under shear and rebuilt at rest. Such time-dependent shear thinning properties increased significantly with the concentration of MWCNTs, as measured by the area of hysteresis loops. In contrast, INFs with long MWCNTs did not show thixotropic properties, except for the sample containing 1 wt % of MWCNTs (Figure 9e); however, they were much weaker compared to INFs with short MWCNTs (Figure 10c). More advanced oscillatory strain sweep tests showed that INFs with short MWCNTs were solid-like materials (the storage modulus prevailed over the loss modulus *G*′ ≫ *G*″) characterized by a relatively narrow range of linear viscoelasticity (LVE) below 1% strain (Figure 10d–f). Enhancing the strain above LVE could disrupt the network structure of INFs for which there was a simple proportionality between elastic strain and stress. In contrast, long MWCNT-based INFs were in fact viscous media with negligible elastic properties (*G*′ ≪ *G*″) (Figure 9f).

## Conclusions

We demonstrate the fundamental role of providing a realistic description of interactions at the ionic liquid-MWCNT interface with its (super)structure, chemistry, and the molecular structure of the continuous phase for efficient heat-transfer fluids. We believe control over the subzipping of nanotube networks in INFs, supported by a careful analysis of the nanotube–IL interface at the molecular level, represents the most efficient tool in the construction of heat-transfer fluids. The established direction could allow engineering INFs from thermoactive components, including multidimensional hybrids such as 1D-CNT/2D-graphene, toward systems of even higher performance, including the low-friction resistance. Our promising results determine the point for further studies on the supramolecular subzipping mechanism, which would govern the “properties-by-design” approach in future customized INFs.

## Supporting Information Available

MD and experimental data (PDF)
