# Supplementary material for: High-Performance Ionanofluids from Subzipped Carbon Nanotube Networks
Source: ACS Appl Mater Interfaces. 2022 Nov 4;14(45):50836–48. doi: 10.1021/acsami.2c14057 (PMC9673059; doi:10.1021/acsami.2c14057)
Supplement: Supplementary file 1 — am2c14057_si_001.pdf [file am2c14057_si_001.pdf]

Supporting Information

High-performance ionanofluids from subzipped carbon nanotube networks

Marzena Dzida[1*], Sławomir Boncel[2,3*], Bertrand Jóźwiak[2,4], Heather F. Greer[5], Mateusz Dulski[6], Łukasz Scheller[1], Adrian Golba[1], Rafał Flamholc[7], Grzegorz Dzido[4], Justyna Dziadosz[1], Anna Kolanowska[2,8], Rafał Jędrysiak[2,3], Anna Blacha[2,3], Krzysztof Cwynar[1], Edward Zorębski[1], Carlos E.S. Bernardes[9], Maria José V. Lourenço[9], Carlos A. Nieto de Castro[9]

[1] University of Silesia in Katowice, Institute of Chemistry, Szkolna 9, 40-006 Katowice, Poland

[2] Silesian University of Technology, Department of Organic Chemistry, Bioorganic Chemistry and Biotechnology, Bolesława Krzywoustego 4, 44-100 Gliwice, Poland

[3] Silesian University of Technology, Centre for Organic and Nanohybrid Electronics, Konarskiego 22B, 44-100 Gliwice, Poland

[4] Silesian University of Technology, Department of Chemical Engineering and Process Design, Marcina Strzody 7, 44-100 Gliwice, Poland

[5] University of Cambridge, Department of Chemistry, Cambridge CB2 1EW, UK

[6] University of Silesia in Katowice, Faculty of Science and Technology, Institute of Materials Science, 75 Pułku Piechoty 1a, 41-500 Chorzów, Poland

[7] Anton Paar Poland, Hołubcowa 123, 02-854 Warsaw, Poland

[8] Silesian University of Technology, Department of Physical Chemistry and Technology of Polymers, Marcina Strzody 9, 44-100 Gliwice, Poland

[9] Centro de Química Estrutural, Institute of Molecular Sciences, Departamento de Química e Bioquímica, Faculdade de Ciências, Universidade de Lisboa, Campo Grande, 1749-016 Lisboa, Portugal

Corresponding Authors

*E-mail: marzena.dzida@us.edu.pl; slawomir.boncel@polsl.pl

Molecular dynamics. The interaction between the carbon surface (CS) and the ionic liquid (IL) was investigated using molecular dynamics (MD) simulation data and the program AGGREGATES[1]. In this characterization, the ions that were in direct contact with the CS were initially determined. For this, the distance between any atom of a cation or anion and any atom of the CS was evaluated. If the obtained value was smaller than the sum of the van der Waals radii of the corresponding atoms, plus 0.05 nm, it was considered that the molecule was in contact with the surface. On average, for the investigated systems, each side of the CS contacts with 16 ion pairs, which implies that, in a given time, 64 ion pairs are in contact with a CS during the simulation (4 surfaces × 16 pairs; see **Figure S1a** for reference). Using this information and the computed system internal energies, the average interaction energy between the IL and the surfaces was also computed. For this, the system's internal energies were compared with those found for a single isolated CS and the neat IL (see details in **Table S1**). The obtained values were then divided by 64, to find an average interaction energy per mole of IL at the surface. The obtained results are given in **Table S1** and represented in **Figure S1b**.

To better understand the causes behind the changes observed in the Raman data, the MD results were also used to investigate the arrangement and conformation of the molecules near the CSs. This study was performed using the AGGREGATES tools, which allow the evaluation of the dihedral angle's distribution, e.g., for molecules located on different sides of the CS surfaces. **Figure S2**, gives the analysis of the $[BMpyr]^+$ alkyl chains conformational changes.

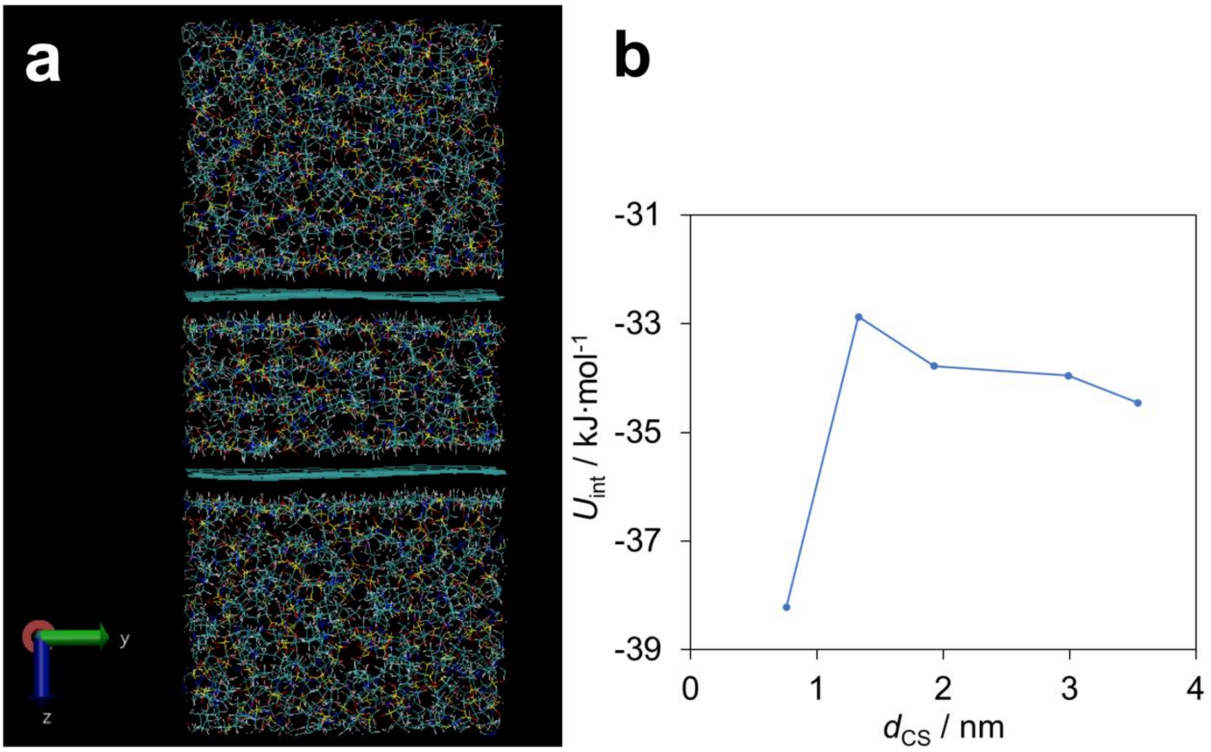

Figure S1. Computations of the CNT-IL interface. (a) Snapshot of the simulation box after optimization. The image corresponds to the box in which the CSs were initially placed at a distance of 2.0 nm. (b) Interaction energy U_{int} between CSs and the IL as a function of the distance d_{CS} between two adjacent carbon structures.

Table S1. Details of the simulation boxes used in the molecular dynamics simulations performed in this work to investigate the interaction between [BMpyr][NTf$_2$] and the carbon surfaces (CS). *d* is the final equilibrium distance between the two graphene sheets, U_{box} is the average system internal energy of the simulation boxes, and U_{int} is the interaction energy between the graphene and the IL. Also given in the table are the final simulation box dimensions and the number of IL pairs placed between and outside the CS.

d (nm)	Box size (nm)	Nº IL (between)	Nº IL (outside)	U_{box} (kJ mol^{-1})	U_{int} (kJ mol^{-1}) [a]

0.76	3.96 × 3.86 × 8.63	16	234	38,155	−38.2
1.33	3.96 × 3.86 × 8.67	30	220	38,497	−32.9
1.93	3.96 × 3.86 × 8.65	57	193	38,439	−33.8
2.99	3.96 × 3.86 × 8.68	75	175	38,428	−34.0
3.54	3.96 × 3.86 × 8.66	100	150	38,396	−34.5
IL [b]	4.95 × 4.95 × 4.95	0	250	28,751	−
CS [c]	3.96 × 3.86 × 10.0	0	0	5,925	−

[a] Interaction energy computed as $U_{int} = (U_{box} - U_{IL} - 2 \cdot U_{CS}) / 64$, where U_{box} is the internal energy of the system under investigation, and U_{IL} and U_{CS} are the internal energy of a simulation box containing 250 IL pairs and one isolated CS, respectively. In the previous equation, 64 represents the average number of IL pairs that are in contact with the two CSs during the simulation (16 pairs on each of the 2 sides of the two surfaces). [b] Simulation results for a simulation box containing 250 ion pairs. [c] Simulation results for a simulation box containing one graphene sheet.

On average, for the investigated system, each side of the CS contacts with 16 ion pairs. This conclusion was, therefore, used to normalize the obtained results into interaction internal energy per mole of IL at the surface. The obtained values are given in **Table S1** and represented in **Figure S1b**. To better understand the causes behind the changes observed in the Raman data, the MD results were also used to investigate the arrangement and conformation of the molecules near the CSs. Three layers of molecules can be defined for the investigated system: layer I and III, with the ions faced to the dispersion bulk, and layer II with the ions located between the two carbon sheets. The results suggest, therefore, that the

cohesive nature of layer II located between the two carbon surfaces leads to a more efficient and stable charge balance of the ions close to the surface. As a result, the obtained structure is more stable (from an internal energy point of view; **Figure S1b**) when compared with arrangements in which the two CNTs are located apart from each other.

Finally, a MD analysis performed on the $[BMpyr]^+$ alkyl chain revealed no conformational change with the distance between the CSs (**Figure S2**), in line with the Raman spectra results.

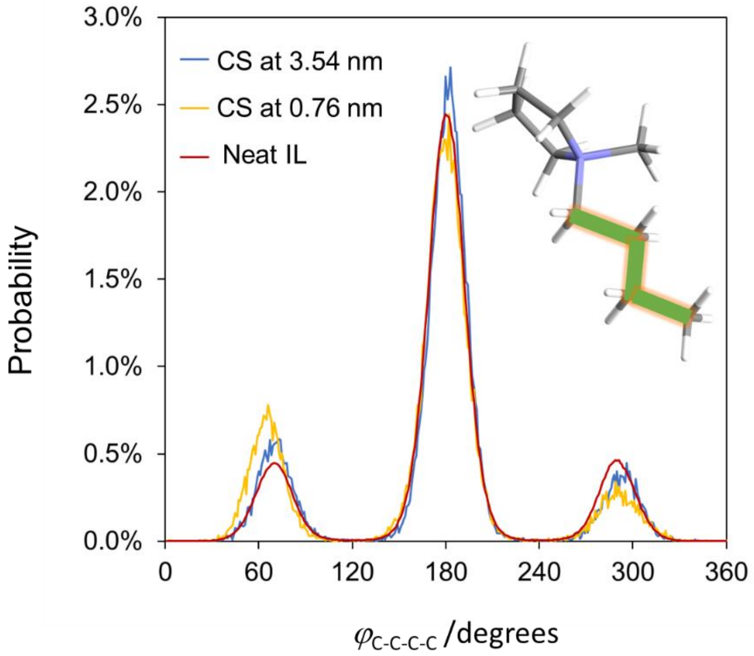

Figure S2. Conformations of the alkyl chain of $[BMpyr]^+$ in: IL versus the IL–CNT interface. Comparison of the distribution of C-C-C-C dihedral angles formed between the carbon atoms of the alkyl chain of $[BMpyr]^+$ (see inset in the plot) in neat IL (red) and located between and in contact with the carbon surfaces at distances of 3.54 nm (blue) and 0.76 nm (yellow).

Table S2. Thermal conductivity λ of [BMpyr][NTf$_2$] and INFs with long and short MWCNTs at 298.15 K.

	λ, *SD* (W m^{-1} K^{-1})					
	[BMpyr][NTf$_2$] + long MWCNTs					
Concentration of long MWCNTs (wt.%)	Series 1	Series 2	Series 3	Mean	*SD*	$\left(\frac{\lambda_{\mathrm{INF}}}{\lambda_{\mathrm{IL}}}-1\right)\cdot 100$ (%)
0	0.119	0.120	0.120	0.120	0.00058	–
0.2	0.130	0.130	0.130	0.130	0	8.6
0.5	0.145	0.145	0.145	0.145	0	21
0.75	0.157	0.156	0.156	0.156	0.00058	31
1	0.168	0.169	0.169	0.169	0.00058	41
	[BMpyr][NTf$_2$] + short MWCNTs					
Concentration of short MWCNTs (wt.%)	Series 1	Series 2	Series 3	Mean	*SD*	$\left(\frac{\lambda_{\mathrm{INF}}}{\lambda_{\mathrm{IL}}}-1\right)\cdot 100$ (%)
0	0.119	0.120	0.120	0.120	0.00058	–
0.2	0.124	0.124	0.124	0.124	0	3.3
0.5	0.130	0.130	0.130	0.130	0	8.3
0.75	0.132	0.132	0.132	0.132	0	10
1	0.137	0.137	0.137	0.137	0	14

SD - standard deviation

References

(1) Bernardes, C. E. S. AGGREGATES: Finding Structures in Simulation Results of Solutions. *Journal of Computational Chemistry* **2017**, *38* (10), 753–765. https://doi.org/10.1002/jcc.24735.
